# Atypical manifestation of late onset limb girdle muscular dystrophy presenting with recurrent falling and shoulder dysfunction: a case report

**DOI:** 10.1186/1757-1626-1-402

**Published:** 2008-12-16

**Authors:** Markus Dietmar Schofer, Thilo Patzer, Markus Quante

**Affiliations:** 1 Department of Orthopaedics, University Hospital Marburg, Marburg, Germany; 2 Department of Spine Surgery, Neustadt General Hospital, Neustadt, Germany

## Abstract

**Introduction:**

Myopathies can be sub-classified into congenital, hereditary, mitochondrial, and secondary myopathies.

Congenital myopathies are usually diagnosed post partal or in early childhood. Manifestation in adolescence is uncommon and most cases occur as sporadic mutations. Therefore, there is a risk of under diagnosing this disease in middle-aged patients showing pain, dysfunction, recurrent trauma or falls, where muscle atrophy is seen as a secondary injury.

**Case presentation:**

Our report is about a 54 year old Caucasian woman with an extended history of pain, loss of function and weakness in her right shoulder. The clinical picture showed a frozen right shoulder. The main finding was a marked limb-muscle atrophy of both delta- und biceps-muscles and a rotator cuff tear that had developed over years. Previous medical consultations attributed the atrophy to recurrent falls, shoulder dysfunction and pain. Conservative treatment (analgesics, physiotherapy, training) had failed.

The familiar anamnesis was free of any neurological diseases or other genetic diseases.

MRI showed a sub-total proximal muscular limb atrophy and a rotator cuff tear in both shoulders. An incision-biopsy of the right delta- and biceps-muscle revealed a chronical myopathy. The level of creatinkinasis was expected to be high but measurements showed values only slightly above normal. Immunohistochemistry, eventually revealed a mild form of LGMD (type 2I). Due to the pattern of symptoms and diagnostic results we described the case as atypical LGMD.

**Conclusion:**

Our case presents a phenotype of a late onset of limb girdle muscular dystrophy syndrome associated with shoulder pain and dysfunction and recurrent falls. This kind of disease is not very common. In particular, muscle atrophy in the elderly is generally seen as a secondary injury. This case should remind us of the importance of a differential diagnosis of a late onset of muscular dystrophy-syndrome in the elderly, since an early diagnosis offers more treatment options, therefore preventing a rapid progression.

## Introduction

Muscular atrophy, occurring very often in connection with bone and joint diseases, is very common in the elderly and can have different causes.

A main cause is the reduced use of muscles or muscle groups, for example around a painful joint. Nerve injury or dysfunction causes neurogenic atrophy to the related muscles. Inflammation can also cause muscle atrophy as well as other secondary disorders of muscles, following general organic diseases like metabolic, endocrine, ischemic, vascular, toxic and paraneoplastic myopathies.

Primary myopathies can be classified in congenital, hereditary, mitochondrial, and progressive muscular dystrophy-syndromes [1-3].

Congenital myopathies are usually diagnosed post partal or in early childhood. Manifestation in adolescence is uncommon [1]. Therefore there is a risk of underdiagnosing this differential diagnosis, if the atrophy is hidden or explained by other symptoms. Pain, recurrent falling, and dysfunction may mislead physicians to interpret muscle atrophy as a secondary symptom.

This rare case of a very late diagnosed myopathy highlights the importance of differential diagnosis. Regarding the simple diagnostic steps (clinical picture, MRI, serum CK, biopsy) myopathy should always be kept in mind by all medical departments treating bone and joint diseases.

## Case presentation

The 54 year old Caucasian woman, who came to our clinic for the first time in September 2006, complained of pain, loss of function and weakness in her right shoulder. She had suffered from these symptoms for years. Conservative treatment by analgesics and physiotherapy showed little or no benefit. Her main reason for coming to our hospital was an increasing loss of function of her right shoulder. In addition she reported several unexplained falls but no feeling of waddling gait and hypotonia. She had osteoporotic vertebral compression fractures at thoracic level 5 and 6. The corresponding thoracic gibbus was compensated by hyperlordosis of the lumbar spine. After a traffic accident in 2004, she was treated conservatively for a fracture of the right scapula and the sternum. She sometimes felt dysaesthesia in both hands. She casually referred to progressive muscle atrophy in both shoulders and upper arms, which other doctors had assigned to previous trauma and inactivity. The familiar anamnesis of her two sons was negative, without signs of neurological or systemic diseases. There was no intellectual impairment or facial weakness.

The patient's clinical examination showed no signs of cardiac or pulmonary impairment. There was a frozen right shoulder, a pronounced isolated limb-muscle atrophy of the delta- und biceps-muscle on both sides and a rotator cuff tear of the supraspinatus and infraspinatus muscle on the right side 1, 2 and 3. There were no signs of muscle impairment in the hip or thigh. The clinical neurological examination revealed a normal result. Walking analysis showed no waddling gait.

In view of the pronounced muscle atrophy of both shoulders, that was progressive over years, despite physiotherapy, a myopathy was suspected although it seemed unlikely in a middle-aged patient.

**Figure 1:**
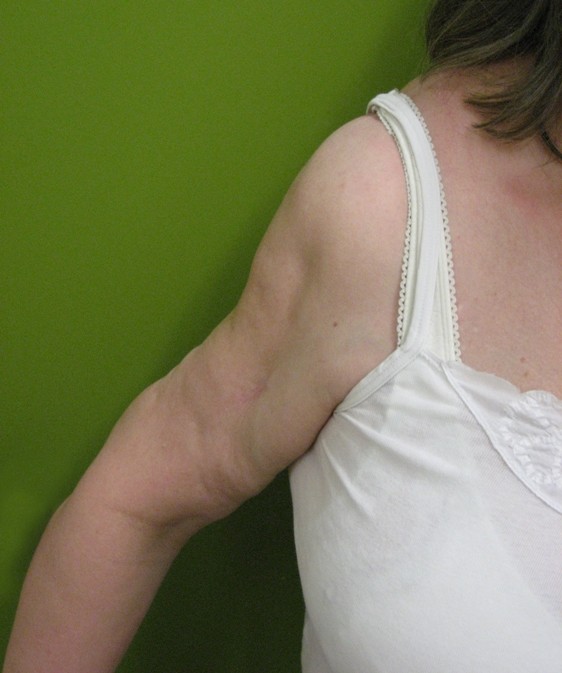
Anterolateral view to the right arm of the patient. The scar results from the incision biopsy. Look at the obvious atrophy of delta muscle.

**Figure 2:**
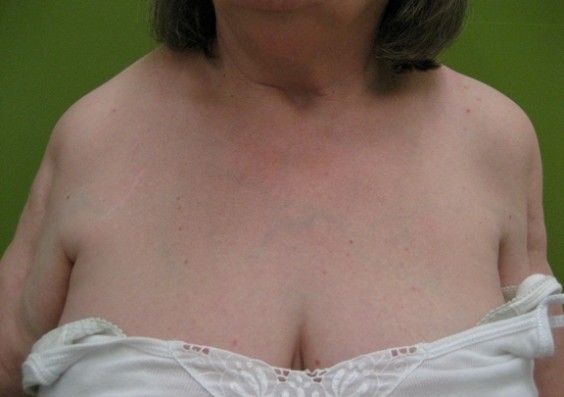
Frontal view of the patient with a manifest reduction of upper arm muscles.

**Figure 3:**
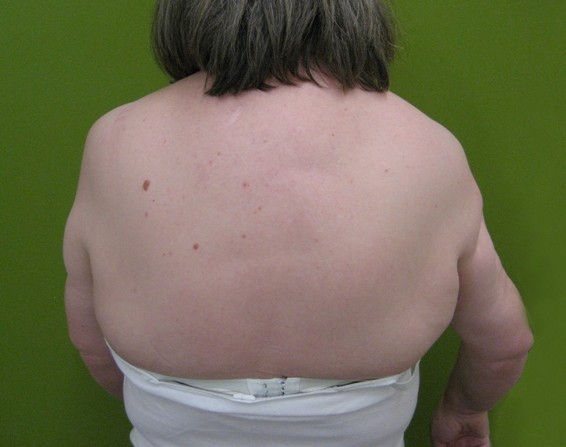
The dorsal view also shows the marked atrophy of the upper arms.

Contrary to the expected high increase of the level of serum creatinkinasis, the level of serum creatinkinasis was only slightly above normal (186 U/l). The exact pattern [1] of muscle atrophy was detected by a MRI [2] of both shoulders which showed a fatty muscular atrophy of the delta- und biceps-muscle on both sides and a rotator cuff tear on the right.

Despite the slightly increased serum creatinkinasis and the symmetric atrophy of identical muscles pointing to a muscular dystrophy of the limb girdle type [1], the diagnosis needed further examination using light microscopy and immunohistochemistry of affected muscles. Therefore we performed an incision biopsy of the right delta- and biceps-muscle to ensure diagnosis. The different samples were kept in formaline, in kryo-fixation, and before being embedded in plastic, in glutaraldehyde.

The results of the immuno-histological (Table 1) analysis matched a chronical myopathy with related endo-, peri- and epimysial fibrosis. Trauma and inflammation were excluded as causes.

**Table 1:** Results of light microscopy and immunohistochemistry

Toluidin-blue and HE-staining	Focally accentuated chronic myopathy with endo-, peri- and epimyseal fibrosis, reduced fiber thickness and augmentation of endomysial connective tissue
Trichrome-staining	Focal subsarcolemmal fuchsinophily, centralised cores

PAS-staining	Regular content of muscle-glycogene

Elastica-von-gieson and oil-red-staining	No amyloid

NADH-TR-enzymeimmunohistochemistry	Normal forming of groups of type I and II muscles, predominance of type I muscle fibers

Immunohistochemistry for myofiber-ATPasis (3 diff. pH's)	Mainly type-I-muscle-fibers/also type-II muscle-fibers had central encymatic defects

Succinatedehydrogenasis and cytochrome-c-oxidasis activity	Normal

Lead-citrate and uranyl-acetate cuts	Muscle-fibers with subsarcolemmal deposited triglycerides and dilatated vacuoles, internal structure partially loosened

Antibodies anti-dystrophine-epitopes 1, 2 and 3 and anti-ubiquitine, merosin, desmine and alpha-sarcoglycane	Regular colouring

Antibodies anti LCS, CD 3 and CD 20	Normal number of mononuclear cells

Further analysis (western blot) revealed an abnormal and reduced laminin α2 chain and an abnormal reduced α-dystroglycan. These findings allow a diagnosis of LGMD2I, where a secondary reduction of laminin-a2 on immunolabelling was detected in most cases and a reduction in α-dystroglycan may also be seen.

We discussed treatment options with the patient. Beside a conservative treatment with muscle training a cortisone therapy was recommended to prevent or slow down further progression. The patient refused a cortisone treatment due to the risk of adverse effects.

## Discussion

The typical pattern of a symmetric muscular atrophy of both shoulder girdles as well as the immuno-histochemical analyses point to a muscle-dystrophic process. Based on our analyses we think that we found a case of a late onset and mild form of LGMD2I. The patient's history makes this case worth publishing. Even though she only showed some symptoms of LGMD, the clinical pattern of muscle atrophy is typical. The immunohistochemistrial changes are also seen in the rarer conditions LGMD2K, 2L and 2N reflecting the common pathological feature of a loss of glycosylation of alpha dystroglycan in this group [2].

The appearance of the disease in this case has to be discussed.

The onset of LGMD 2I occurs between the age of 0.5 to 27 years (61% < 5) [1]. The age of onset may vary both between and within subtypes and even between patients with the same mutation. There is an early onset in teenage years and a later onset, with slower progress and only some non-ambulant patients (28%), in the 4^th ^to 6^th ^decades [4,5]. Late manifestation has also been described in LGMD 1A, 1C and is very unlikely in the other forms of LGMD [2,6-8].

Respiratory involvement and failure is common in LGMD 2I, as well as cardiac involvement [2], but was not seen in our case. Overnight pulse oximetry, if the FVC is <60%, annual influenza vaccination and prompt treatment of respiratory infections are recommended [1.6,9] with respiratory involvement.

Patients can develop secondary contractures in LGMD 2I, whereas other forms of LGMD have no contractures or primary contractures (LGMD 1B, 2A). The finding of a frozen shoulder may partially be an effect of the disease but it can also be caused by an independent process.

Serum CK mirrors the amount of muscle destruction. Depending on the type of LGMD, CK is not elevated or only slightly elevated (< 5× upper limit of normal at LGMD 1A, 1B), elevated (5× to 10× upper limit of normal at LGMD 1C, 2A, 2C-F, 2I) or highly elevated (>10× upper limit of normal at LGMD 2B and also 1C, 2A, 2C-F, 2I) [10]. Hence, we expected a high elevation of CK in our patient. The comparably marginal increase of CK may reflect a low disease activity. It has to be underlined that low or normal CK levels do not exclude a LGMD [1,2].

Leading clinical symptoms of LGMD are weakness with a waddling gait, difficulty in stair-climbing and hypotonia. Weakness is more proximal than distal in arms and legs. Thigh adductors, psoas and quadriceps muscle are usually affected in the lower limbs. The periscapular muscles, deltoid, biceps and triceps muscle are typically involved in the upper limbs. There was a mild facial weakness in some patients described [1,2,5,6]. In our case, we found typical symptoms that had developed over years. Maybe the coincidence of several falls and shoulder pain prompted the therapists to classify the muscle atrophy as secondary. A muscle disease or muscle atrophy was not considered up to that point.

Further diagnostics using DNA analysis were discussed. DNA analysis, aimed at providing confirmation of mutation in the affected gene(s), is necessary to be able to offer carrier or pre-symptomatic testing to other family members [2,11,12]. We discussed this topic with the patient and her sons but they declined further DNA analysis. An early diagnosis, even in mild forms with the late onset, may improve chances of treatment and outcome. However, there are no established drug treatments for the LGMD. Creatine monohydrate and co-enzyme Q10 (ubiquinone) were tested but not under controlled conditions [2,3] and corticosteroids have been used empirically in some patients with LGMD2C-F with reported improvement [13]. Preclinical studies are working on gene transfer and stem cell transplantation [14].

## Conclusion

Late onset and mild manifestation of LGMD 2I in the middle-aged patient is uncommon. Irrespective of the typical pattern of muscle atrophy, there is a high risk of underdiagnosis, particularly if other parameters like serum CK levels and clinical signs (intellectual impairment, facial weakness) are inconspicuous. The potential benefit of an early therapy LGMD in the elderly, has to be kept in mind.

## List of abbreviations

CD: cluster of differentiation; CK: serum-creatinkinasis; DNA: deoxyribonucleic acid; HE-staining: Hematoxylin and Eosin staining; kDA: kilo Dalton; LCS: leukaemic cell surface; LGMD: limb girdle muscular dystrophy-syndrome; MRI: magnetic resonance imaging; NADH-TR: Nicotinamide adenine dinucleotide dehydrogenase tetrazolium reductase; PAS-staining: Periodic acid-Schiff staining.

## Consent

Written informed consent was obtained from the patient for publication of this case report and accompanying images. A copy of the written consent is available for review by the Editor-in-Chief of this journal.

## Competing interests

The authors declare that they have no competing interests.

## Authors' contributions

SM: Manuscript, clinical evaluation, surgery, TP: Manuscript, clinical evaluation. QM: Manuscript, literature review, all the authors read and approved the final manuscript.
